# 454-Pyrosequencing Reveals Variable Fungal Diversity Across Farming Systems

**DOI:** 10.3389/fpls.2016.00314

**Published:** 2016-03-14

**Authors:** Elham A. Kazeeroni, Abdullah M. Al-Sadi

**Affiliations:** Department of Crop Sciences, College of Agricultural and Marine Sciences, Sultan Qaboos UniversitySeeb, Oman

**Keywords:** oasis farming, soil-borne fungi, pesticides, organic matter, fungal communities, population dynamics

## Abstract

Oasis farming system is common in some parts of the world, especially in the Arabian Peninsula and several African countries. In Oman, the farming system in the majority of farms follows a semi-oasis farming (SOF) system, which is characterized by growing multiple crops mainly for home consumption, but also for local market. This study was conducted to investigate fungal diversity using pyrosequencing approach in soils from a farm utilizing a SOF system which is cultivated with date palms, acid limes and cucumbers. Fungal diversity from this farm was compared to that from an organic farm (OR) growing cucumbers and tomatoes. Fungal diversity was found to be variable among different crops in the same farm. The observed OTUs, Chao1 richness estimates and Shannon diversity values indicated that soils from date palms and acid limes have higher fungal diversity compared to soil from cucumbers (SOF). In addition, they also indicated that the level of fungal diversity is higher in the rhizosphere of cucumbers grown in OR compared to SOF. *Ascomycota* was the most dominant phylum in most of the samples from the OR and SOF farms. Other dominant phyla are *Microsporidia*, *Chytridiomycota*, and *Basidiomycota*. The differential level of fungal diversity within the SOF could be related to the variation in the cultural practices employed for each crop.

## Introduction

Soil is a reservoir of thousands of fungal and bacterial species that play important roles in natural and managed agricultural soils (Abed et al., 2013; Al-Sadi et al., 2015b; Kaisermann et al., 2015; Tardy et al., 2015). Fungi are the most dominant eukaryotic species in terms of biomass in soil. Fungi play important roles as decomposers, nutrient cyclers, soil aggregators, pathogens, and mycorrhizal symbionts (Guo et al., 2015; Thomson et al., 2015; Stott and Taylor, 2016).

Changes in land use and agricultural practices have resulted in reduction in soil quality, fertility and productivity (Cherubin et al., 2015; Price et al., 2015). The productivity and health of soils rely to some extent on the processes of soil microbial communities (Guo et al., 2015; Heilmann-Clausen et al., 2015; Stott and Taylor, 2016). The high use of inorganic fertilizers and pesticides can affect soil microbial populations and result in reduction of microbial diversity or changes in microbial communities (Esmaeili Taheri et al., 2015; Filimon et al., 2015; Pose-Juan et al., 2015; Rangel et al., 2015).

Oasis farming system has been a common practice in the Arabian Peninsula over 1000s of years. The system is characterized by growing several crops, mainly date palms, around a water resource. The water resource, date palms and other crops used to provide a pleasant environment for people, where several people belonging to the same or different tribes used to live together (Kharusi and Salman, 2015). This system has been developed into a semi-oasis, conventional farming system in most growing areas in the Arabian Peninsula, especially in Oman, where farmers continued to grow multiple crops in plots which are irrigated by flowing water or underground water extracted from wells (Al-Sadi et al., 2013; Yaish and Kumar, 2015). Most farms in Oman these days have at least two or more of the following crops: date palms, acid limes, mangoes and cucumbers (Al-Sadi et al., 2013). Animal manures are commonly used in these farms, especially for date palms and citrus (Al-Sadi et al., 2011, 2015b). However, potting media, composts and several inorganic fertilizers are introduced and used for vegetable crops, mainly cucumbers (Al-Mazroui and Al-Sadi, 2015). In addition, the infection of cucumbers by fungal pathogenic fungi necessitates frequent applications of systemic and contact fungicides (Al-Sadi, 2012). On the other hand, there is a shift toward the use organic farming (OR) in some farms in the country, but the growth rate of this sector is very slow (Al-Mazroui and Al-Sadi, 2015).

Previous studies have shown that the population size and structure of soil flora and fauna can be affected by several factors, including the cultivation techniques, plant species, and the application of organic and inorganic fertilizers and pesticides (DeAngelis et al., 2015; Matsushita et al., 2015; Tardy et al., 2015; Van Geel et al., 2015; Coleman-Derr et al., 2016). However, little information is available concerning the effect of cultivation systems on fungal diversity in this part of the world. In addition, little is known about the level of fungal diversity between different crops in the same farm.

This study was conducted to investigate fungal diversity in semi-oasis and OR systems. Specific objectives are: (1) to investigate the level of fungal diversity in a semi-oasis farming (SOF) system, and (2) to characterize the extent of fungal diversity in relation to OR and in the rhizosphere of different crops. Investigations into the dynamics of microbial populations in farming systems could help provide information about the health status of soils in SOF systems, and improve the shift toward more organic-dependent commercial farming systems in the developing countries.

## Materials and Methods

### Collection of Samples

The study investigated fungal diversity in a SOF system in Oman in comparison to OR. The experiment was located in one OR and one SOF. Details on the location, crops, and weather conditions are presented in **Table 1**.

**Table 1:** Characteristics of samples and sampling locations.

Sample code	Farming system	Crop	Sampling site	Geographical location	Temperature range (°C)	Annual rain fall (mm)
OR-CU	Organic	Cucumber	Farm #1	N 23° 40.81668′, E 58° 10.95′	15.9–42.9	7 mm
OR-TO	Organic	Tomato	Farm #1	N 23° 40.81668′, E 58° 10.95′	15.9–42.9	7 mm
SOF-CU	Semi-oasis	Cucumber	Farm #2	N 23° 41.02458′, E 57° 54.29364′	13.4–43.3	21 mm
SOF-AL	Semi-oasis	Acid lime	Farm #2	N 23° 41.02458′, E 57° 54.29364′	13.4–43.3	21 mm
SOF-DP	Semi-oasis	Date palm	Farm #2	N 23° 41.02458′, E 57° 54.29364′	13.4–43.3	21 mm


Soil samples were collected during September–November, 2013, from the rhizosphere of cucumber and tomato in the OR and from cucumber, acid lime, and date palm in SOF. The rhizosphere soil in this study is considered soil which is within the 0–3 cm distance from plant roots. Cucumber and tomato have been grown in the OR for at least the last 8 years. Date palms and acid limes grown in the SOF were 9–12 years old. They have been fertilized using animal manure and have never received any fungicide treatment in the soil. Cucumbers in the SOF have been fertilized using inorganic fertilizers and animal manures. Cucumber soils from the SOF farm received several mefenoxam, hymexazol and Thiophanate-methyl treatments over the last 5 years.

Each soil sample was approximately 1kg collected from three locations in the top 5–15 cm depth near (0–3 cm) the active feeder roots of each crop. Soil samples were collected from the rhizosphere of three randomly selected crops of each crop species grown in a different plot within a farm. The soil samples were kept in sterile plastic bags and then transferred to the Plant Pathology Research Lab, SQU, Oman. Each sample was subjected to chemical and physical analysis. Soil for DNA analysis was ground with liquid nitrogen and then kept at -80°C until DNA extraction.

### Soil Physicochemical Properties

Soil samples were air-dried, ground, and then passed through a 2-mm sieve to remove roots and plant debris. After that the sieved soil was stored in plastic tubes until analysis. Various physicochemical parameters of each soil sample were determined. The texture of the soil was determined by using hydrometer test (Gee and Bauder, 1986). Electrical conductivity (EC) and pH were determined by using EC and pH meters (Zhang et al., 2005). Potassium (K) contents were measured with flame photometric method (Sheerwood 450 flame photometer) while the concentration of phosphorus (P) was determined by using Inductively Coupled Plasma (Perkin Elmer, USA). Total inorganic carbon (TIC) and total organic carbon (TOC) was analyzed by using Total Organic Carbon analyzer (TOC-V, Shimadzu, Japan). Analysis of nitrogen was done by mixing 0.5 g soil sample with one tablet of Kjeltab catalyst in 10 ml of sulfuric acid, followed by heating at 420°C for 20–30 min. Then the solution was allowed to cool, followed by analysis of total nitrogen using Kjeltec Analyser (FOSS TECATOR, Sweden).

### Pyrosequencing Analyses

DNA was extracted from soil samples according to the protocol of Volossiouk et al. (1995), following some modifications. Soil was ground with liquid nitrogen for 5 min and then 0.05 g of each sample was transferred to 1.5 μl eppendorf tube. Then, 125 μl of skimmed milk was added and the mixture was incubated in an oven at 65°C for 1 h. The mixture was centrifuged and the supernatant was transferred into a new eppendorf tube. After that 500 μl SDS extraction buffer (0.3 % SDS, 140 mM NaCl, 50 mM NaAc, pH 5.1) was added to the supernatant, followed by the addition of one volume of phenol: chloroform: isoamyl alcohol (25:24:1). The suspension was centrifuged and the supernatant was transferred to a new eppendorf tube. Then, 0.6 volumes of isopropanol and 10 μl of NaAc was added to the supernatant, incubated overnight at -20°C and then centrifuged. The pellet was washed with 600 μl ethanol, dried and finally suspended in TE buffer. The quality and quantity of DNA was assessed by using a Nano drop spectrophotometer (Thermo Scientific, USA).

DNA extraction was carried out from the three replicate samples collected from each crop. The three replicate DNA samples of each crop were then composted together. The primers ITS1F and ITS2aR were used for the amplification of ITS2 region. The samples were submitted to the Research and Testing Laboratory (RTL, Lubbock, TX, USA) for tag-encoded 454-pyrosequencing (Dowd et al., 2008a,b).

The obtained sequences which are less than 300 bp were excluded from further analysis and the rest were checked for high quality using RDP ver 9 (Cole et al., 2009). The low quality ends and tags were removed and were checked for chimers using UCHIME chimera detection software (Edgar et al., 2011). The resulting sequences were analyzed using a BLASTn.NET algorithm by comparison to high quality sequences from NCBI and the outputs were validated based on taxonomic distance methods (Dowd et al., 2005, 2008a,b). Out of 1980–14189 raw reads for the seven samples, 1230–11829 reads were obtained after filtering at 97% similarity threshold.

Further analysis of pyrosequencing data was carried out using the R software (R Development Core Team, 2011). A rarefaction curve plot was generated showing the number of OTUs versus the number of sequences. Richness was estimated using the Chao1 richness estimator using the formula: S_chao1_ = S_obs_ + n_1_ (n_1_-1)/2 (n_2_ + 1), where S_obs_ is the number of observed species/OTUs and n*i* is the number of OTUs with abundance *i*. Shannon Diversity was calculated using the formula H′ = -Σpiln(pi), where *P_i_* is the proportion of phylotypes, and *i* is the total number of all phylotypes in the sample. UniFrac and Bray–Curtis distances were calculated using the phyloseq package in R. Principal Coordinate Analyses (PCoA) were conducted and plotted from weighted and unweighted UniFrac distances and Bray–Curtis distances. Heatmap of relative abundances of the most dominant fungal genera was generated and the samples were sorted based on Bray–Curtis, weighted UniFrac and unweighted UniFrac distances.

### Statistical Analysis

Tukey’s Studentized range test (SAS, SAS Institute Inc., USA) was used to examine differences among soils from different cropping systems. Correlation analysis was also conducted using SAS.

## Results

### Soil Physicochemical Properties

Soils from different crops and farming systems showed variation in their physicochemical properties (**Table 2**). The OR soils were loamy sandy, while soils from SOF were found sandy and loamy sandy. The pH of most soils was found to be neutral to alkaline (pH = 7.7–8.4), while EC ranged from 1 to 10.54 mS. The level of inorganic carbon was found to be significantly higher in soils from SOF compared to OR, while organic carbon was found to be significantly higher in the soil from date palms compared to all other soils (*P* < 0.05). The levels of N, P, and K were variable among different farming systems and crops (**Table 2**).

**Table 2:** Physicochemical analysis of soil samples.

Code	Soil texture	pH	EC (mS)	%TIC	%TOC	%N	P (mg/kg)	K (mg/kg)
OR-CU	Loamy sand	8.4^a^	0.99^c^	1.06^b^	1.90^b^	0.06ˆb	3.71^a^	7.46ˆb
OR-TO	Loamy sand	8.4^a^	1.21^c^	0.02^c^	2.89^b^	0.05ˆb	3.25^a^	24.57ˆab
SOF-CU	Loamy sand	7.7^b^	4.98^b^	5.65^a^	2.30^b^	0.02ˆc	4.48^a^	58.37ˆa
SOF-AL	Sandy	8.1^ab^	2.92^c^	5.84^a^	2.27^b^	0.08b	0.09^b^	26.77ˆab
SOF-DP	Sandy	8.2^ab^	2.20^c^	4.98^a^	4.93^a^	0.12ˆa	0.51^b^	15.36ˆb


*EC, electrical conductivity; TIC, total inorganic carbon; TOC, total organic carbon; N, nitrogen; P, phosphorus; and K, potassium. Values with the same letter in the same column are not significantly different from each other at *P* < 0.05 (Tukey’s Studentized Range test, SAS).*

### Relationship between Crops and Fungal Diversity

**FIGURE 1:**
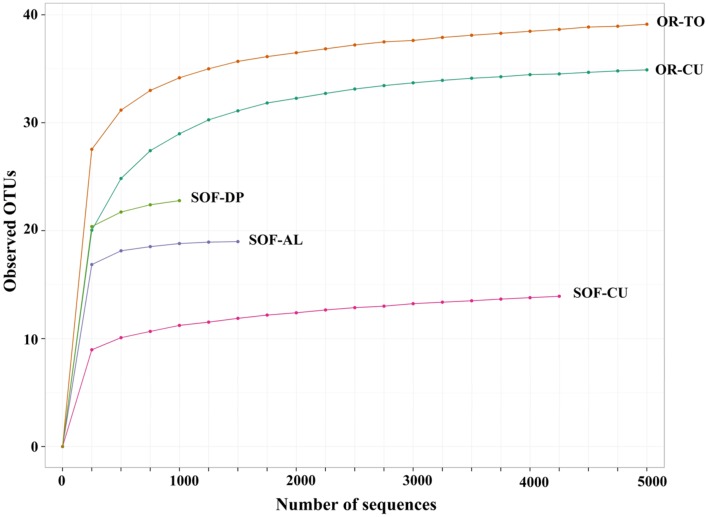
**Rarefaction curve of observed OTUs of two soil samples obtained from tomato (OR-TO) and cucumber (OR-CU) grown in organic farm and three soil samples obtained cucumber (SOF-CU), acid lime (SOF-AL) and date palm (SOF-DP) grown in a semi-oasis farm**.

**FIGURE 2:**
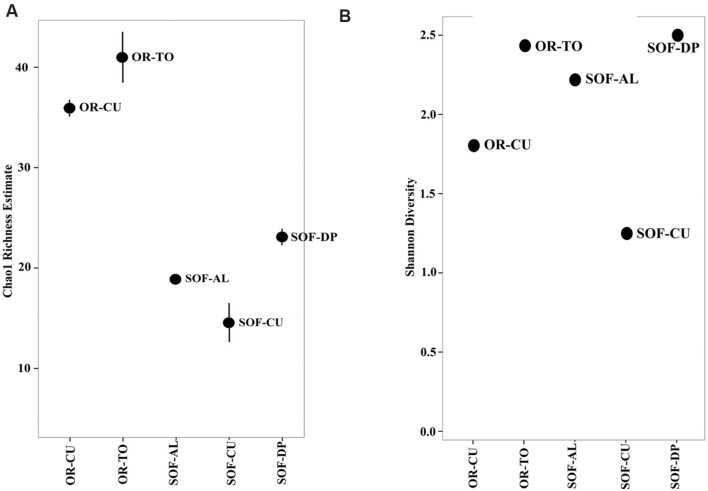
**Chao1 richness **(A)** and Shannon diversity **(B)** within the total microbiome data of five soil samples obtained from organic farm (OR) and semi-oasis farm (SOF).** Sample codes are described in **Table 1**.

Analysis of fungal diversity in the SOF, which is growing date palm, acid lime and cucumber, showed the presence of differential diversity among the three crops. Chao1 richness estimates showed that a total of 23, 19 and 14 OTUs were detected in the rhizosphere from the three crops, respectively (**Figures 1** and **2**). Shannon diversity estimates showed that date palm has the highest value which is 2.50, followed by acid lime (2.22) and cucumber (1.25) (**Figure 2**).

**FIGURE 3:**
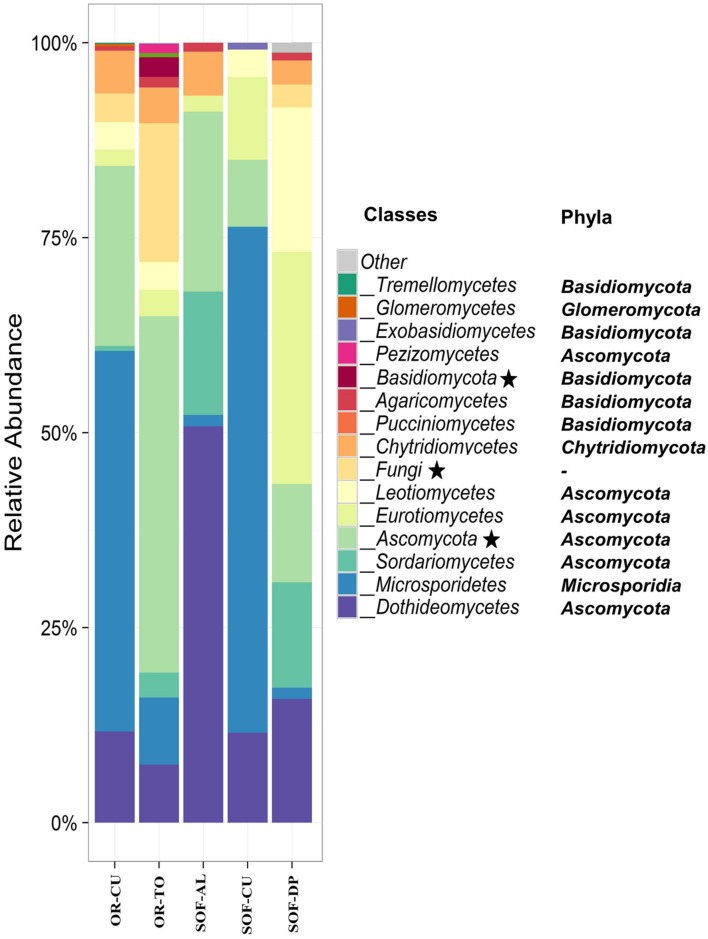
**Class-level relative abundance of fungal communities in organic (OR) and semi-oasis farming (SOF) systems.** Asterisks in the legend indicate taxonomic units that could not be resolved to the class level. The phyla of the different classes are indicated on the figure. Sample codes are described in **Table 1**.

**FIGURE 4:**
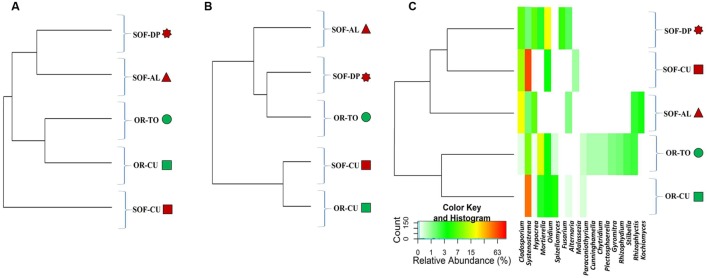
**Heatmap of relative abundances of the 18 dominant fungal genera in the five samples.** Samples are sorted based on Bray–Curtis distances **(A)**, weighted Unifrac distances **(B)** and unweighted Unifrac distances **(C)**. The colored shapes next to sample codes indicate the source of soils (green for organic and dark red for semi-oasis). The fungal genera are described in **(C)** while sample codes are described in **Table 1**.

*Ascomycota* was the most dominant phylum, present in soil from the three crops. The other dominant phyla were *Microsporidia*, *Basidiomycota*, and *Chytridomycota* (**Figure 3**). The classes *Dothideomycetes, Microsporidetes*, and *Sordariomycetes* were the most common in soils from the three crops (**Figure 3**). Other common classes included *Eurotiomycetes, Leotiomycetes*, and *Chytridiomycetes*. Acid lime shared seven common classes with date palm, while cucumber shared five common classes with date palm. The number of shared classes between acid lime and cucumber were four. *Cladosporium* and *Systenostrema* were detected in soils of the three crops, while the other fungal species were detected in soil of one to two crops. *Systenostrema* had high abundance in soil from cucumber (**Figure 4**).

### Fungal Diversity in Organic versus Semi-Oasis Farms

A higher level of fungal diversity was observed in the rhizosphere of cucumbers grown in OR compared to SOF. The Shannon and Chao1 richness values were 1.80 and 36 for cucumber soil from OR compared to 1.25 and 14 for soil from SOF, respectively (**Figure 2**). Tomato grown in the OR also had relatively high Shannon diversity and richness values (**Figure 2**).

*Ascomycota* phylum was the most dominant phylum in most of the soil samples cultivated with cucumber in the OR and SOF as well as in the organic tomato. Other phyla included *Microsporidia*, *Chytridiomycota*, and *Basidiomycota.* Our results revealed that *Microsporidetes* was the main class in cucumber grown in OR and SOF (**Figure 3**).

**FIGURE 5:**
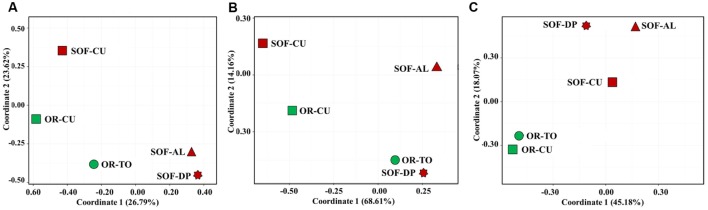
**Principal coordinates analysis of fungal diversity in five soil samples based on Bray–Curtis distances **(A)**, weighted Unifrac distances **(B)** and unweighted Unifrac distances **(C)**.** The green and dark red colors indicate organic and semi-oasis farming systems, respectively. Sample codes are described in **Table 1**.

Analysis of fungal diversity in the OR and SOF soils using unweighted UniFrac distances showed separation of ORs from SOFs (**Figures 4** and **5**). On the other hand, Weighted UniFrac separated cucumber grown in OR and SOF from other crops while Bray–Curtis also separated OR cucumbers and tomato from the others, with close relationship with SOF-DP/AL (**Figures 4** and **5**). Further statistical analyses could not be done due to the use of one technical and one biological replicate for each sample. No correlation was found between diversity estimates and soil physicochemical properties (*P* > 0.05).

## Discussion

Semi-oasis farming system in Oman has been found to harbor differential level of fungal diversity. The level of fungal diversity was higher from soils cultivated with date palms and citrus compared to soils cultivated with cucumbers. This is the first 454-pyrosequncing-based study which addressed fungal diversity in SOF. The lower level of fungal diversity in the rhizosphere of cucumber could be related to the use of fungicides in this crop. Cucumbers are heavily affected by wilt and other root diseases, which necessitate frequent applications of mefenoxam, thiophanate-methyl, hymexazol and other fungicides (Al-Sadi, 2012; Al-Sadi et al., 2012, 2015a). It is therefore likely that fungicide applications could have resulted in lowering fungal populations in the rhizosphere of cucumbers. On the other hand, fungicides’ use for date palms and acid lime in Oman is minimal or does not exist at all in many farms, because of the limited fungal diseases which affect the two crops, especially date palms. In addition, tillage, which has been reported to decrease fungal diversity (Tardy et al., 2015), is frequently practiced for soils growing cucumbers, which is not the case for date palms and acid limes.

Previous studies have shown that microbial diversity increases significantly in farms which depend on organic manure as compared to chemical fertilization (Qiu et al., 2012; Zhang et al., 2012). Organic manures are known to harbor high fungal diversity (Al-Mazroui and Al-Sadi, 2015; Al-Sadi et al., 2015b). The application of animal manures instead of mineral fertilizers to date palms and acid limes could have contributed to increasing the diversity of fungi in soil from these crops. It also appears that the type of crops grown have influenced the level of fungal diversity and the interaction between these crops and fungi (Badri and Vivanco, 2009; Sugiyama et al., 2010).

The number of OTUs, Shannon diversity and Chao1 estimate revealed that the level of fungal diversity in soil from organically grown cucumber was higher compared to cucumber grown inorganically. This supports findings from previous studies about the higher fungal diversity in organic soils (Sugiyama et al., 2010). This could be related in part to the use of organic manures as well as that chemical fungicides are not used in soils in OR. In addition, organic composts which are applied frequently to these farms are usually treated with some fungal species for biocontrol purposes, which may explain the higher level of fungal diversity in these farms (Al-Sadi et al., 2015b; Kohler et al., 2015; On et al., 2015).

Unweighted UniFrac, weighted UniFrac and Bray–Curtis analyses of fungal diversity showed clustering of the different soil samples into different groups as influenced either by the faming systems and/or by plant species. Unweighted UniFrac analysis separated soils according to the farming systems, i.e., different farming systems made a qualitative difference in fungal diversity. However, weighted UniFrac showed clustering based on plant species, suggesting some dominant taxa in cucumber, which seems to be the Systenostrema (Microsporidetes). Bray–Curtis does not take into account taxonomic relatedness between OTUs, while UniFrac does. This consideration resulted in closer clustering between OR-TO with OR-CU, SOF-AL and SOF-DP, but did not affect similarity between SOF-CU and OR-CU (**Figure 5A**). This shows that the OTUs that made the difference between OR-TO/CU and SOF-AL/DP detected in Bray-Curtis are somehow phylogenetically related. Also the overall variances explained by coordinate 1 and 2 is higher in weighted UniFrac (∼83%), compared to unweighted (∼63%) and Bray-Curtis (∼50%). This may suggest that plant species and/or associated cultural practices determine the fungal community structure, although the faming systems have strong impact on the qualitative structure. In another study on *Agave* species, Coleman-Derr et al. (2016) suggested that biogeography of the host species is the major determinant of fungal microbiome.

*Ascomycota* phylum was the most dominant phylum in most of the samples from OR and SOF. This phylum is widespread in different soils around the world (Qiu et al., 2012; Abed et al., 2013; Al-Sadi et al., 2015b). This is mainly because *Ascomycota* contains several pigmented species which usually tolerate higher temperatures and because many species of this produce abundant spores (Abed et al., 2013). *Microsporidia*, *Chytridiomycota*, and *Basidiomycota* phyla were also common in some farm soils. Differences in the species composition between the different soils could be related to the different growing systems, cultural practices and different crops.

Physicochemical analysis of soils showed variations in soil pH, carbon and nutrients from one soil to the other. The levels of organic carbon were the same or lower in the organic soils compared to soils from semi-oasis system. Previous studies have shown that organic systems do not always increase organic matter in the soil compared to conventional systems (Li et al., 2012). The slightly lower soil pH in SOF compared to OR is in agreement with previous studies (Li et al., 2012). Soil pH and nutrients are usually affected by vegetation, soil type, carbon and nitrogen (Barak et al., 1997; Kuramae et al., 2012).

## Conclusion

Our study showed the presence of differential levels of fungal diversity that were suggested to be associated with farming systems and plant species or cultural practices. The SOF system seems to have high level of fungal diversity, especially under crops which are grown in a semi-organic way. This is expected to be reflected on soil health and quality especially if the fungal communities are of the plant growth promoting or disease suppressive species. The trend toward production of cucumber in these systems appears to have impact on the fungal diversity levels, possibly due to the heavy use of fungicides. The current shift toward the use of pesticides in SOF systems should take into consideration the negative side of chemicals on diversity of the beneficial fungi.

## Author Contributions

AA-S planned for research, analyzed data, wrote paper. EK did the experiments, analyzed data and helped in writing paper.

## Conflict of Interest Statement

The authors declare that the research was conducted in the absence of any commercial or financial relationships that could be construed as a potential conflict of interest.

## References

[B1] Abed R. M. M. Al-Sadi A. M. Al-Shihi M. A. Al-Hinai S. Robinson M. D. (2013). Diversity of free-living and lichenized fungal communities in biological desert crusts of the Sultanate of Oman and their role in improving soil properties. *Soil Biol. Biochem.* 57 695–705. 10.1016/j.soilbio.2012.07.023

[B2] Al-Mazroui S. S. Al-Sadi A. M. (2015). Highly variable fungal diversity and the occurrence of potentially plant pathogenic fungi in potting media, organic fertilizers and composts originating from 14 countries. *J. Plant Pathol.* 97 529–534. 10.4454/JPP.V97I3.033

[B3] Al-Sadi A. M. (2012). Efficacy of mefenoxam is affected by a lag period between application and inactivation of *Pythium* species. *Phytopathol. Mediterran.* 51 292–297.

[B4] Al-Sadi A. M. Al-Ghaithi A. G. Al-Balushi Z. M. Al-Jabri A. H. (2012). Analysis of diversity in *Pythium aphanidermatum* populations from a single greenhouse reveals phenotypic and genotypic changes over 2006 to 2011. *Plant Dis.* 96 852–858. 10.1094/PDIS-07-11-0624 30727347

[B5] Al-Sadi A. M. Al-Masoodi R. S. Al-Ismaili M. Al-Mahmooli I. H. (2015a). Population structure and development of resistance to hymexazol among *Fusarium solani* populations from date palm, Citrus and cucumber. *J. Phytopathol.* 163 947–955. 10.1111/jph.12397

[B6] Al-Sadi A. M. Al-Mazroui S. S. Phillips A. (2015b). Evaluation of culture-based techniques and 454 pyrosequencing for the analysis of fungal diversity in potting media and organic fertilizers. *J. Appl. Microbiol.* 119 500–509. 10.1111/jam.12854 25996218

[B7] Al-Sadi A. M. Al-Said F. A. Al-Jabri A. H. Al-Mahmooli I. H. Al-Hinai A. H. de Cock A. W. A. M. (2011). Occurrence and characterization of fungi and oomycetes transmitted via potting mixtures and organic manures. *Crop Prot.* 30 38–44. 10.1016/j.cropro.2010.09.015

[B8] Al-Sadi A. M. Al-Wehaibi A. N. Al-Shariqi R. M. Al-Hammadi M. S. Al-Hosni I. A. Al-Mahmooli I. H. (2013). Population genetic analysis reveals diversity in *Lasiodiplodia* species infecting date palm, Citrus, and mango in Oman and the UAE. *Plant Dis.* 97 1363–1369. 10.1094/PDIS-03-13-0245-RE 30722139

[B9] Badri D. V. Vivanco J. M. (2009). Regulation and function of root exudates. *Plant Cell Environ.* 32 666–681. 10.1111/j.1365-3040.2009.01926.x 19143988

[B10] Barak P. Jobe B. O. Krueger A. R. Peterson L. A. Laird D. A. (1997). Effects of long-term soil acidification due to nitrogen fertilizer inputs in Wisconsin. *Plant Soil* 197 61–69. 10.1023/a:1004297607070

[B11] Cherubin M. R. Franco A. L. C. Cerri C. E. P. Oliveira D. M. S. Davies C. A. Cerri C. C. (2015). Sugarcane expansion in Brazilian tropical soils-Effects of land use change on soil chemical attributes. *Agricult. Ecosyst. Environ.* 211 173–184. 10.1016/j.agee.2015.06.006

[B12] Cole J. R. Wang Q. Cardenas E. Fish J. Chai B. Farris R. J. (2009). The Ribosomal Database Project: improved alignments and new tools for rRNA analysis. *Nucleic Acids Res.* 37 D141–D145. 10.1093/nar/gkn879 19004872 PMC2686447

[B13] Coleman-Derr D. Desgarennes D. Fonseca-Garcia C. Gross S. Clingenpeel S. Woyke T. (2016). Plant compartment and biogeography affect microbiome composition in cultivated and native Agave species. *New Phytol.* 209 798–811. 10.1111/nph.13697 26467257 PMC5057366

[B14] DeAngelis K. M. Pold G. Topçuoglu B. D. van Diepen L. T. A. Varney R. M. Blanchard J. L. (2015). Long-term forest soil warming alters microbial communities in temperate forest soils. *Front. Microbiol.* 6:104 10.3389/fmicb.2015.00104 PMC4327730 25762989

[B15] Dowd S. E. Callaway T. R. Wolcott R. D. Sun Y. McKeehan T. Hagevoort R. G. (2008a). Evaluation of the bacterial diversity in the feces of cattle using 16S rDNA bacterial tag-encoded FLX amplicon pyrosequencing (bTEFAP). *BMC Microbiol.* 8:125 10.1186/1471-2180-8-125 PMC2515157 18652685

[B16] Dowd S. E. Sun Y. Wolcott R. D. Domingo A. Carroll J. A. (2008b). Bacterial tag encoded FLX amplicon pyrosequencing (bTEFAP) for microbiome studies:bacterial diversity in the ileum of newly weaned *Salmonella*-infected pigs. *Foodborne Pathog. Dis.* 5 459–472. 10.1089/fpd.2008.0107 18713063

[B17] Dowd S. E. Zaragoza J. Rodriguez J. R. Oliver M. J. Payton P. R. (2005). Windows.NET network distributed basic local alignment search toolkit (W.ND-BLAST). *BMC Bioinformat.* 6:93 10.1186/1471-2105-6-93 PMC1087835 15819992

[B18] Edgar R. C. Haas B. J. Clemente J. C. Quince C. Knight R. (2011). UCHIME improves sensitivity and speed of chimera detection. *Oxford J. Bioinformat.* 27 2194–2200. 10.1093/bioinformatics/btr381 PMC3150044 21700674

[B19] Esmaeili Taheri A. Hamel C. Gan Y. (2015). Pyrosequencing reveals the impact of foliar fungicide application to chickpea on root fungal communities of durum wheat in subsequent year. *Fungal Ecol.* 15 73–81. 10.1016/j.funeco.2015.03.005

[B20] Filimon M. N. Voia S. O. Popescu R. Dumitrescu G. Ciochina L. P. Mituletu M. (2015). The effect of some insecticides on soil microorganisms based on enzymatic and bacteriological analyses. *Roman. Biotechnolog. Lett.* 20 10439–10447.

[B21] Gee G. W. Bauder J. W. (1986). “Particle size analysis,” in *Methods of Soil Analysis, Part-I, Physical and Mineralogical Methods*, ed. Klute A. (Madison, WI: American Society of Agronomy), 383–411.

[B22] Guo X. Petermann J. S. Schittko C. Wurst S. (2015). Independent role of belowground organisms and plant cultivar diversity in legume-grass communities. *Appl. Soil Ecol.* 95 1–8. 10.1016/j.apsoil.2015.05.010

[B23] Heilmann-Clausen J. Barron E. S. Boddy L. Dahlberg A. Griffith G. W. Nordén J. (2015). A fungal perspective on conservation biology. *Conservation Biol.* 29 61–68. 10.1111/cobi.12388 25185751

[B24] Kaisermann A. Maron P. A. Beaumelle L. Lata J. C. (2015). Fungal communities are more sensitive indicators to non-extreme soil moisture variations than bacterial communities. *Appl. Soil Ecol.* 86 158–164. 10.1016/j.apsoil.2014.10.009

[B25] Kharusi N. S. Salman A. (2015). In search of water: hydrological terms in Oman’s toponyms. *Names* 63 16–29. 10.1179/0027773814Z.00000000094

[B26] Kohler J. Caravaca F. Azcón R. Díaz G. Roldán A. (2015). The combination of compost addition and arbuscular mycorrhizal inoculation produced positive and synergistic effects on the phytomanagement of a semiarid mine tailing. *Sci. Total Environ.* 514 42–48. 10.1016/j.scitotenv.2015.01.085 25659304

[B27] Kuramae E. E. Yergeau E. Wong L. C. Pijl A. S. Van Veen J. A. Kowalchuk G. A. (2012). Soil characteristics more strongly influence soil bacterial communities than land-use type. *FEMS Microbiol. Ecol.* 79 12–24. 10.1111/j.1574-6941.2011.01192.x 22066695

[B28] Li R. Khafipour E. Krause D. O. Entz M. H. de Kievit T. R. Fernando W. G. D. (2012). Pyrosequencing reveals the influence of organic and conventional farming systems on bacterial communities. *PLoS ONE* 7:e51897 10.1371/journal.pone.0051897 PMC3526490 23284808

[B29] Matsushita Y. Bao Z. Kurose D. Okada H. Takemoto S. Sawada A. (2015). Community structure, diversity, and species dominance of bacteria, fungi, and nematodes from naturally and conventionally farmed soil: a case study on Japanese apple orchards. *Organ. Agricult.* 5 11–28. 10.1007/s13165-015-0096-4

[B30] On A. Wong F. Ko Q. Tweddell R. J. Antoun H. Avis T. J. (2015). Antifungal effects of compost tea microorganisms on tomato pathogens. *Biol. Cont.* 80 63–69. 10.1016/j.biocontrol.2014.09.017

[B31] Pose-Juan E. Sánchez-Martín M. J. Herrero-Hernández E. Rodríguez-Cruz M. S. (2015). Application of mesotrione at different doses in an amended soil: dissipation and effect on the soil microbial biomass and activity. *Sci. Total Environ.* 536 31–38. 10.1016/j.scitotenv.2015.07.039 26188530

[B32] Price G. W. Astatkie T. Gillis J. D. Liu K. (2015). Long-term influences on nitrogen dynamics and pH in an acidic sandy soil after single and multi-year applications of alkaline treated biosolids. *Agricult. Ecosys. Environ.* 208 1–11. 10.1016/j.agee.2015.04.010

[B33] Qiu M. Zhang R. Xue C. Zhang S. Li S. Zhang N. (2012). Application of bio-organic fertilizer can control Fusarium wilt of cucumber plants by regulating microbial community of rhizosphere soil. *Biol. Fertil. Soils* 48 807–816. 10.1002/jsfa.5653

[B34] R Development Core Team (2011). *R: A Language and Environment for Statistical Computing*. Vienna: R Foundation for Statistical Computing.

[B35] Rangel D. E. N. Alder-Rangel A. Dadachova E. Finlay R. D. Kupiec M. Dijksterhuis J. (2015). Fungal stress biology: a preface to the fungal stress responses special edition. *Curr. Genet.* 61 231–238. 10.1007/s00294-015-0500-3 26116075

[B36] Stott M. B. Taylor M. W. (2016). Microbial ecology research in New Zealand. *N. Z. J. Ecol.* 40 12–28.

[B37] Sugiyama A. Vivanco J. M. Jayanty S. S. Manter D. K. (2010). Pyrosequencing assessment of soil microbial communities in organic and conventional potato farms. *Plant Dis.* 94 1329–1335. 10.1094/PDIS-02-10-0090 30743621

[B38] Tardy V. Spor A. Mathieu O. Lévèque J. Terrat S. Plassart P. (2015). Shifts in microbial diversity through land use intensity as drivers of carbon mineralization in soil. *Soil Biol. Biochemist.* 90 204–213. 10.1016/j.soilbio.2015.08.010

[B39] Thomson B. C. Tisserant E. Plassart P. Uroz S. Griffiths R. I. Hannula S. E. (2015). Soil conditions and land use intensification effects on soil microbial communities across a range of European field sites. *Soil Biol. Biochemist.* 88 403–413. 10.1016/j.soilbio.2015.06.012

[B40] Van Geel M. Ceustermans A. Van Hemelrijck W. Lievens B. Honnay O. (2015). Decrease in diversity and changes in community composition of arbuscular mycorrhizal fungi in roots of apple trees with increasing orchard management intensity across a regional scale. *Mol. Ecol.* 24 941–952. 10.1111/mec.13079 25586038

[B41] Volossiouk T. Robb E. J. Nazar R. N. (1995). Direct DNA extraction for PCR-mediated assays of soil organisms. *Appl. Environ. Microbiol.* 61 3972–3976. 8526511 10.1128/aem.61.11.3972-3976.1995 PMC167704

[B42] Yaish M. W. Kumar P. P. (2015). Salt tolerance research in date palm tree (Phoenix dactylifera L.), past, present, and future perspectives. *Front. Plant Sci.* 6:348 10.3389/fpls.2015.00348 PMC4434913 26042137

[B43] Zhang H. Schroder L. L. Pittman J. J. Wang J. J. Payton M. E. (2005). Soil salinity using saturated paste and 1;1 soil and water extracts. *Soil Sci. Soc. Am. J.* 69 1146–1151. 10.2136/sssaj2004.0267

[B44] Zhang Q. C. Shamsi I. H. Xu D. T. Wang G. H. Lin X. Y. Jilani G. (2012). Chemical fertilizer and organic manure inputs in soil exhibit a vice versa pattern of microbial community structure. *Appl. Soil Ecol.* 57 1–8. 10.1016/j.apsoil.2012.02.012

